# Effects of eyeshades in sleep quality and pain after surgery in school-age children with supracondylar humeral fractures

**DOI:** 10.3389/fped.2023.1192217

**Published:** 2023-09-04

**Authors:** Xiang-xuan Wang, Kai-nan Lin, Wen-chen Xu, Hui Chen, Hao-qi Cai

**Affiliations:** ^1^ Department of Pediatric Orthopedics, Fujian Children’s Hospital (Fujian Branch of Shanghai Children’s Medical Center), Fuzhou, China; ^2^ College of Clinical Medicine for Obstetrics and Gynecology and Pediatrics, Fujian Medical University, Fuzhou, China; ^3^ Department of Orthopedics Shanghai Children’s Medical Center, Shanghai Jiao Tong University School of Medicine, Shanghai, China

**Keywords:** eyeshades, supracondylar fracture of humerus, postoperative, school-age children, sleep quality, pain

## Abstract

**Objective:**

This study aimed to explore the effects of eye masks on the sleep quality and pain of children over 5 years old with humeral supracondylar fracture after surgery.

**Methods:**

Fifty children with humeral supracondylar fracture who underwent closed reduction and percutaneous pinning (CRPP) in the Pediatric orthopaedic Department of a provincial hospital in China from February 2020 to December 2021 were selected. The children were randomly divided into the experimental group (*n* = 25) and the control group (*n* = 25). Children in the control group were given routine sleep care, and the children in the experimental group were given a sleep intervention with eye masks for three nights after surgery. The Pittsburgh Sleep Quality Index was used to evaluate the sleep quality of the children. The Children’s Pain Behaviour Scale was used to evaluate the pain of the children.

**Results:**

After three nights of receiving the eye mask intervention, the children in the experimental group had significantly lower sleep quality scores than those in the control group; the difference was statistically significant (*p* < 0.05), and the children in the experimental group had higher sleep quality. The experimental group’s pain scores were significantly lower than the control group’s, and the difference was statistically significant (*p* < 0.05), and the children in the experimental group experienced less post-operative pain.

**Conclusions:**

Eye masks are a simple, safe and economical intervention, that is beneficial for improving the sleep quality and reducing pain in children over 5 years old with humeral supracondylar fracture after closed reduction and percutaneous pinning. It can be used as a reference and basis for clinical pain relief and sleep quality after surgery for supracondylar fractures of the humerus in children.

## 1. Introduction

Supracondylar fractures of the humerus are the most common upper extremity fractures in children and are associated with localized swelling and pain with limited mobility (1, 2). Forms of treatment for these fractures include casting, traction, open reduction, and closed reduction with percutaneous pinning (CRPP) (3). Sleep is an essential human need, and getting enough sleep might help patients recover faster (4). Sleep difficulties are frequent postsurgery complaints for people with humeral supracondylar fractures. Environmental variables, as well as postoperative pain and discomfort, all contribute to the development of sleep disorders. For children with supracondylar humerus fractures, the early postoperative pain and discomfort associated with cast immobilization often affects the quality of their sleep.

According to research (5), when sleep is disrupted, the sensation of pain increases, wound healing is delayed, and the pathological process that promotes the development of chronic pain can continue unabated. A decreased pain threshold and hyperalgesia are also linked to sleep deprivation (6). Previous research has found that eyeshades can help patients improve their sleep quality (7, 8).

Given these research findings, this study aimed to investigate the impact of eyeshades on sleep quality and pain after surgery and CRPP in school-age children with humeral supracondylar fracture and provide specific guidance for postoperative rehabilitation of these young patients.

## 2. Materials and methods

After approval by our ethics committee, the clinical data of 54 school-age children with supracondylar humerus fractures who underwent closed reduction with percutaneous pinning at our institution from February 2020 to December 2022 were collected. Four patients withdrew from this study due to noncooperation after surgery. The remaining 50 children were randomly divided into the experimental group (*n* = 25) and the control group (*n* = 25).

Inclusion criteria were as follows: (1) children between the ages of 7 and 12 years with supracondylar humerus fractures who underwent closed reduction with percutaneous pinning and could cooperate with the medical staff to participate in this study; (2) normal mental status and no intellectual disability; (3) no serious complications in the perioperative period; and (4) children with parents or guardians who agreed to participation in this study and signed the informed consent form.

Exclusion criteria were as follows: Patients who had (1) sleep disorders and/or insomnia before admission; (2) chronic pain; (3) serious complications in the perioperative period; (4) communication difficulties; and (5) parents or guardians who refused to consent to their child’s participation in this study.

Before the operation, we randomly divided the patients into experimental group and control group by random method (random number generated by computer). To reduce our research selection bias, three physicians agreed on the inclusion and exclusion of patients in both the professionals and control group.

Gender, age, weight, type of fracture, time from trauma to surgery, whether ice was applied before surgery, time of operation and the number of Kirschner pins used in both groups were recorded. All children with supracondylar humerus fractures were operated on by the same paediatric orthopaedic surgeon and underwent closed reduction with percutaneous pinning, with the limb fixed in a functional elbow position in a long-arm tubular cast.

Description of research sites: Our wards include many single rooms with just one patient per room, and during the COVID-19 pandemic, we only allowed one accompanying family member to stay. To eliminate bias in the results caused by the number of patients in the room and other noises in the room, all patients in this study were admitted to these single rooms.

### 2.1. Sample size calculation method

In our study, the sample size calculation was performed using Altman’s nomogram, and considering the studies of Dai (9) as examples, the calculated sample size required was 20 with power = 90%, β = 10% and α = 5%. Based on an estimated 10% attrition rate, we selected 25 subjects for each group and then randomly divided the subjects into the two groups.

### 2.2. Sleep quality assessment

This study used the Pittsburgh Sleep Quality Index (10) to assess sleep quality in patients with supracondylar fractures of the humerus after CRPP. The Pittsburgh Sleep Quality Index, developed in 1989 by Dr Buysse, a psychiatrist at the University of Pittsburgh, USA (11), has seven components: subjective sleep quality, sleep latency, sleep length, habitual sleep efficiency, sleep disruptions, use of sleep medications, and daytime dysfunction. Each component of the Pittsburgh Sleep Quality Index is scored on a 0–3 scale, for a total score ranging from 0 to 21 points. The lower the score, the worse the sleep quality.

### 2.3. Behavioural pain assessment

We used the Face, Legs, Activity, Cry, Consolability (FLACC) Pain Assessment Scale, which is straightforward, well-validated, and minimally impacted by interpersonal variance for children aged 0–18 years. The FLACC Pain Assessment Scale has five items, each scored on a 0–2 scale, for a total score of 0–10 points (12). The clinical staff observed and scored the child for 2 min on the above five items to assess the child’s pain level, and the study results showed that a score of ≤3 points indicated mild pain or no pain, 4–7 points indicated moderate pain, and 8–10 points indicated severe pain (13).

### 2.4. Postoperative interventions

After preoperative randomization, all children underwent CRPP by the same highly qualified specialist, and all were externally fixed with a tubular cast of the affected limb postoperatively. Before the start of the experiment, the Pittsburgh Sleep Quality Index score and the Face, Legs, Activity, Cry, Consolability (FLACC) score were measured in the two groups of patients. Children in the control group were given routine postoperative sleep care, including reducing noise and light stimulation and minimizing night-time treatment procedures. In addition to receiving the same routine postoperative sleep care, children in the test group were issued eye masks, given instructions on how to wear them, and directed to wear their mask between 21:00 and 06:00 daily. In the experimental group after 3 consecutive nights of mask wear, the Pittsburgh Sleep Quality Index scores and the Face, Legs, Activity, Cry, and Consolability (FLACC) scores of the two groups of children were reassessed.

### 2.5. Data collection and aggregation

By searching hospital records, screened 54 eligible patients were screened for the study, four of whom withdrew from the study due to noncooperation after surgery. For the remaining 50 patients, clinical information including sex, age, height, weight, subtype of supracondylar humeral fracture, and duration of surgery was collected. A trained paediatric orthopaedic nurse who served as an observer for this study obtained sleep quality and pain scores for children in both groups immediately before the start of the trial and after the experimental group had worn the eyeshades for 3 nights.

### 2.6. Statistical analysis

SPSS version 22.0 was used for statistical analysis. The chi–square test was used to compare qualitative data between groups. The *t*-test was used to assess differences between the groups in sleep quality and pain scores, which were reported as means and standard deviations, and *P* < 0.05 indicated that the difference was significant.

## 3. Results

The demographic data of the two groups are shown in Table 1. There is no significant differences between the two groups in terms of age, sex, height, weight, time of surgery or fracture classification (*p* > 0.05), indicating that the two groups were comparable.

**Table 1:** Comparison of the general data of the two groups of children.

Variable	The experimental group (*n* = 25)	The control group (*n* = 25)	*P* value
Gender			
Female	9	11	0.895
Age (years)	5.8 ± 1.6	6.1 ± 1.4	0.938
Weight (kg)	20.3 ± 3.4	21.6 ± 4.1	0.882
Gartland typology			0.539
II	7	8	
III	13	14	
IV	5	3	
Time from injury to operation (h)	23.5 ± 3.5	19.6 ± 4.6	0.91
Pre-operative icing	17	15	0.556
Operation time (min)	40 ± 12	38 ± 14	0.887
Kirschner wires Number			0.547
2	19	18	
3	6	7	

Table 2 shows a comparison of sleep scores between the two groups of children. Before the intervention, there was no statistically significant difference in the Pittsburgh Sleep Quality Index scores between the two patient groups (*p* > 0.05). After the intervention, children in the experimental group had significantly lower scores in the seven domains of the Pittsburgh Sleep Quality Index and lower total Pittsburgh Sleep Quality Index scores than before the intervention (*p* < 0.05) and significantly lower post-intervention scores than children in the control group (*p* < 0.05). Children in the control group also had significantly lower sleep latency and sleep duration scores after the intervention period compared to before the intervention (*p* < 0.05) even though they did not wear a sleep mask. However, the control group showed no significant changes in subjective sleep quality, habitual sleep efficiency, sleep disturbance, use of sleep medications, daytime dysfunction, or total Pittsburgh Sleep Quality Index scores after the intervention period compared to before the intervention (*p* > 0.05).

**Table 2:** Comparison of sleep quality scores between the two groups of children before and after the eye mask intervention.

Item	EG (B)	EG (A)	CG (B)	CG (A)
Subjective sleep quality	2.11 ± 0.32	1.21 ± 0.23*^,^^#^	1.89 ± 0.43	1.85 ± 0.22
Sleep latency	2.01 ± 0.55	1.25 ± 0.18*^,^^#^	1.98 ± 0.28	1.87 ± 0.19
Sleep duration	1.98 ± 0.34	1.30 ± 0.26*^,^^#^	1.79 ± 0.34	1.39 ± 0.18*
Habitual sleep efficiency	1.87 ± 0.33	1.33 ± 0.17*^,^^#^	1.79 ± 0.32	1.61 ± 0.23
Sleep disturbances	1.84 ± 0.42	1.23 ± 0.24*^,^^#^	1.90 ± 0.24	1.39 ± 0.26*
Use of sleeping medications	1.79 ± 0.52	1.29 ± 0.21*^,^^#^	1.87 ± 0.28	1.66 ± 0.23
Daytime dysfunction	1.95 ± 0.36	1.32 ± 0.27*^,^^#^	1.93 ± 0.41	1.75 ± 0.32
Total score	13.55 ± 0.87	8.93 ± 0.66*^,^^#^	13.15 ± 0.69	11.22 ± 0.47

EG, experimental group; CG, control group.

B, before the intervention; A, after the intervention.

* *p* < 0.05 compared with before the intervention.

^#^ *p* < 0.05 compared with the control group.

As shown in Table 3, there was no significant difference in FLACC (Face, Legs, Activity, Cry, Consolability) scores between the two groups of children before the intervention (*p* > 0.05). After the intervention, the FLACC scores of children in the experimental group were significantly lower than those before the intervention and lower than those in the control group (*p* < 0.05). There was no significant difference in FLACC scores in the control group before and after the intervention period (*p* > 0.05).

**Table 3:** Comparison of FLACC scores between the two groups of children before and after intervention.

Item	The experimental group	The control group	Nominal *P* value
Before the intervention	5.9 ± 1.1	6.1 ± 0.8	0.959
After the intervention	4.3 ± 0.8*	5.7 ± 1.2	0.046

* *p* < 0.05 compared with before the intervention.

## 4. Discussion

Supracondylar humerus fractures are the most common type of fracture in children. Treatment for these fractures includes casting, traction, open reduction, and closed reduction with percutaneous pinning (1). Children with supracondylar humerus fractures usually require 4–6 weeks of cast immobilization and a long recovery period after surgery (1). For this group of children, pain and discomfort are often evident early in the postoperative period, which can affect their sleep quality. Adequate sleep is essential for recovery after fracture surgery. When sleep is disrupted or restricted, the body’s perception of pain increases, tissue repair is delayed, and the pathological process that leads to the development of chronic pain continues indefinitely (14). Therefore, identifying factors that can affect sleep is a key component in the postoperative care of children with these fractures. The purpose of this study was to clarify the effect of eye mask use on sleep quality and pain intensity in children who undergo surgery for supracondylar humeral fractures. We hypothesized that postoperative eye mask use was more effective than usual care in improving sleep quality and reducing pain in these young patients.

Early studies (15) have shown that the addition of eyeshades improves sleep quality in patients compared to usual care, including studies conducted with cardiac patients. There have been no reports on the effects of eye masks on postoperative sleep and pain in children with fractures. The mechanism by which the use of eye masks affects sleep quality is not yet clear and may be related to increased melatonin activity. Since melatonin plays a key role in inducing sleep (16), darkness at night can induce an increase in melatonin secretion in the body, which results in improved sleep quality. The stimulation of artificial light at night during hospitalization can affect the secretion of melatonin in the body, thus disrupting the patient’s sleep. The use of an eye mask can block out light and reduce the disturbance, which helps to create a dark environment conducive to sleep (17).

This study has shown that the use of an eyeshade has a significant role in reducing postoperative pain in humeral supracondylar fractures in children. The results indicate that the use of an eye patch is a safe, effective and economical adjunct to routine sleep care that reduces postoperative pain in children with supracondylar humerus fractures who undergo CRPP. Krause’s research (18) showed that sleep deprivation increased an individual’s experience of pain and significantly lowered the pain threshold of the patients studied. Another experimental study in healthy adults showed that relative to baseline levels, sleep deprivation significantly decreased mechanical pain thresholds, and both REM sleep and slow wave sleep interruptions tended to decrease mechanical pain thresholds (19). In our study, after the intevention, the facial, leg, activity, crying, and comfortability scores of the children in the experimental group who wore sleep masks were significantly lower than those before the intervention and lower than those of the children in the control group, with a statistically significant difference (*p* < 0.05). These results suggest that the eye mask intervention was effective in relieving children’s postoperative pain; therefore, it facilitated their postoperative recovery.

This research has some limitations. First, this was a retrospective study rather than a randomized controlled trial, and some bias in the selection of medical record data is possible. Second, this study was conducted in a single location with a small sample size. The Pittsburgh Sleep Quality Index is somewhat subjective and prone to bias in terms of sleep quality scores, and a more objective method of sleep monitoring could be used later. Third, as there are no previous studies on the effect of eye shields on postoperative sleep in children with supracondylar humerus fractures, the sample size in this study was calculated by referring to the literature on postoperative cardiac surgery, which may not have been appropriate for our study. Fourth, it can be used as a reference and basis for clinical pain relief and sleep quality after surgery for supracondylar fractures of the humerus in school-age children. To confirm the study’s results, prospective, multicentre, randomized, large sample size studies will be needed.

In conclusion, eyeshades are beneficial as a simple, cost-effective intervention to improve the quality of sleep and reduce postoperative pain in school-age children who undergo surgery for supracondylar humerus fractures. The results suggest that the use of eyeshades for this purpose is worth considering and possibly promoting in clinical practice.

## Data Availability

The raw data supporting the conclusions of this article will be made available by the authors, without undue reservation.
